# The Moving Lines on Electron Spectra as Charge Reflexes on Non-equilibrium States of Nanostructured Surfaces

**DOI:** 10.1186/s11671-016-1395-8

**Published:** 2016-04-16

**Authors:** Oleg A. Mishchuk

**Affiliations:** Tribology and Surface Chemistry Laboratory, Ukrainian Scientific and Research Institute for Crude Oil Refining Industry “MASMA”, Palladin Av., 46, 03142 Kyiv, Ukraine

**Keywords:** Electron induced spectrum, Charge, Moving line, Electron stage, Nanostructured surface, Cluster, Inceptive adsorption layer, Non-equilibrium state, Ionization

## Abstract

The experimental results present the phenomenon of moving lines on electron spectra which are linked spatially and in time with the localization and durability of the processes of new surface producing in folds and grain boundaries. This effect was also realized for a thin-layer composite “organic on metal films on dielectric substrate” in modeling non-equilibrium conditions which are created by the intensive electron beam pulse impact. It was found that the nature of the inceptive adsorption layer, in addition to the metal film, determines the initial positions of moving lines on the spectra. The main accents in these investigations were in observations of appearance of the moving lines, dynamics of their displacements on the spectra, final stages when these lines vanished, and finding the general regularities between the spontaneous and induced events.

## Review

Non-stable surface layers for various materials during their structure transformations may be sources of some interesting effects, especially at new grain boundaries [1–12]. One of the effects is the soliton-like “moving line” (ML) which appears on an electron-induced Auger spectrum simultaneously with well-known “stationary” spectral lines.

These MLs, which is studied in this work, are not chaotic disturbances in a spectral background level. In common, there are next characters of their appearance.

- MLs are induced by electron probes and not by positive ion probes.
- The first appearance of MLs often takes place near Auger spectral lines of some elements in a form of satellites.
- Then, these lines move along the spectrum within a range of several tens and even hundreds of electron volts during a long enough time. Simultaneously, the positions of the spectral lines for various chemical elements remain stable.
- Under electron probe acting, these lines displace in the direction of lower energies of the spectrum. The rate of this movement is increased with electron current density.
- Under additional action of positive ion probes, these lines either slow down their movement or begin to displace in the direction of higher energies of the spectrum.
- MLs appear on electron spectra only for some microregions of the sample surface, rather for highly dispersive zones or surface folds which have a relatively large local quasi-static charge. In the same time, they do not appear for other surface regions.
- After vanishing of the MLs, the marked surface regions will not become a source of this phenomenon again. Because the spontaneous appearance of ML for surfaces of polycrystalline solids is rare, their prognostication is problematic today but actual for nanostructured surfaces.

Consequently, the preliminary analysis shows that in many different experimental cases various MLs are of the same long-term dynamic spectral feature in secondary electron distributions and may be a reflection of discrete electron stages of surface atoms at summary impacts of changeable local electrical charges in surface layers which have non-equilibrium structure. But more detailed causes and especially the physical nature of MLs remain debatable.

The aims of this work were in research and comparison of properties of spontaneous and specially induced MLs and, as a result, in forecast analysis of the nature of this phenomenon.

The objects of three different types were studied by the methods of SEM [13]; electron-induced Auger electron spectroscopy (AES) [14]; electron ionization spectroscopy (EIS) [15]; and also argon ion sputtering of surface layers using the JEOL JAMP-10S Auger microprobe in the regime of the *E*d*N*(*E*)/d*E* mode by a cylindrical mirror modulation method for analogue differentiation of direct spectra, at a normal direction of an incident electron beam (energy *E*_e_ = 5 keV, diameter less 1 μm) to a sample surface. Other parameters were depended from the objects.

For numerical analysis of spectral lines’ shape, all differential spectra of secondary electrons were integrated in an identical manner considering 1/*E* factor and subtractions of a general background distribution curve for inelastic electron scattering and Auger line shapes from direct spectra (i.e., SBL procedure) and using cos^2^*x* extrapolation for component peaks in procedure of fitting. These operations significantly reduced the impact of spectral noise without mathematical smoothing.

### An Incident of Low-Intensity Moving Line

Let use the steel friction surface after mild wear as the first object of the investigations. A well-known four-ball kinematic scheme (Hertzian point contact [16], balls with diameter 12.7 mm produced from the steel 100Cr6 of martensitic class, HRC 62) and 1 % solution of zinc dithiophosphate Zn[SPS(OR)_2_]_2_ in n-paraffin (hexadecane С_16_Н_34_) were taken in a ground of tribology experiment realized with Falex FB-AW Test Machine.

**Fig. 1:**
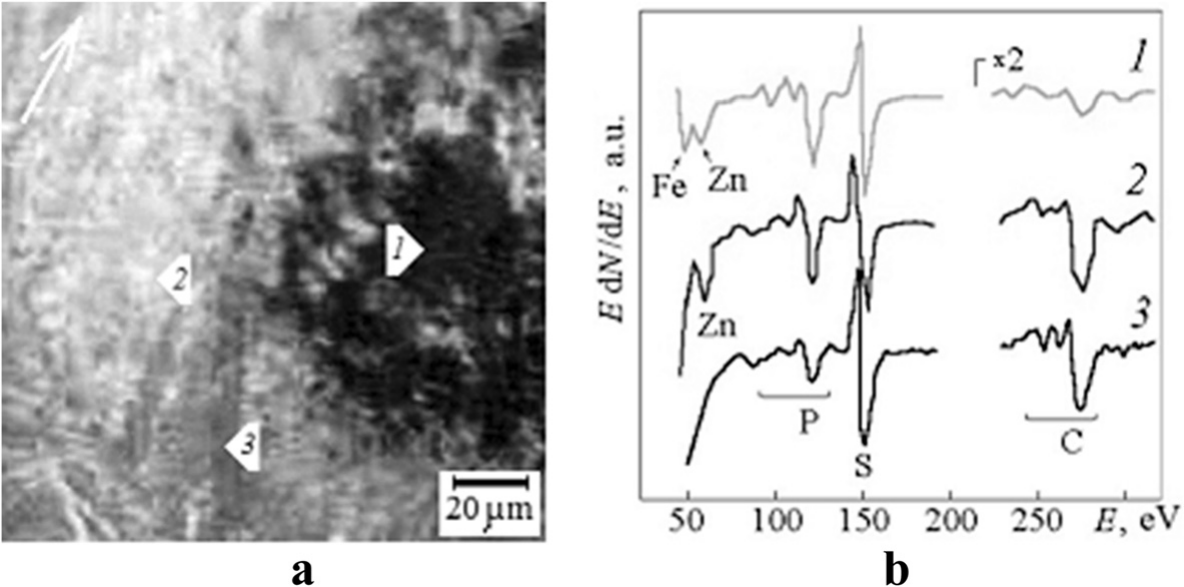
Various types of surface tribofilms for worn Hertzian point contact. **a** SEI contrast of the different surface tribofilms after friction of steel balls in 1 % solution of Zn[SPS(OR)_2_]_2_; the *pointer* indicates the direction of friction force. **b** Fine structure of Auger spectral lines for surface microzones 1–3

A friction process leads to multi-vector (for various surface zones) large strain deformations of steel, as launch factor, simultaneously with adsorption of the Zn[SPS(OR)_2_]_2_ molecules and, as a result, causes the different dynamic relaxation of surface martensitic structure (tribochemical transformations) and a creation of various gradient nanograin architectures of steel surface layers [17]. Analysis of the worn surface of a steel stationary ball after friction (Fig. 1a) shows different states of surface tribofilms in various regions. For microzones 1–3, the spectra in Fig. 1b illustrate this conclusion too. The properties of these microzones were studied carefully in [12].

A legible and durable charge surface contrast (Fig. 1a) which appeared in the influence of an electron beam is a specific sign of a difference of the tribofilms. The diameter of a region with a dark phase contrast coincides with the size of central elastic compression zone [16] for two non-worn steel balls. In comparison with initial elastic Hertzian contact, microzones 2 and 3 were located in the peripheral region of tangential stretching.

Now, it is important that in conditions of analysis of a compositional depth profile, i.e., during argon ion sputtering of the whole region illustrated in Fig. 1a, the line of the ML type which changed its position on the spectra step by step in terms of ion irradiation appeared only in microzone 2. What are the peculiarities of this microzone? It was located in the region with a relatively bright phase contrast (Fig. 1a). Compared to other regions, there were maximal concentration of zinc (up to 21 at. %) and minimal concentrations of iron and oxygen. Due to saturation of surface layers by zinc in microzone 2, the general formation mechanism for transitional non-stable carbides (Fig. 1b, spectrum 3) of M2X-like type was blocked and surface structures of the М6Х type were formed in diffusive under-surface layers [12]. And the phenomenon of ML took place in these layers.

The surface layers of a sample were studied using step-by-step argon ion sputtering with an effective rate of 3 nm/min which provided an ion gun with differential vacuum pumping. The angle between the incident ion beam and the average surface normal was 60°. The energy, current density, and diameter of the ion beam were 2 keV, nearly 0.5 μA/mm^2^, and 2 mm, respectively. In this regime, the vacuum level around the sample was better for 3 × 10^−5^ Pa.

The electron spectra are registered between the steps of sputtering without pauses at the following additional parameters: modulation *M* = 4 eV, primary electron beam density *i* = 0.5 A/mm^2^, *ΔЕ*/*Е* = 0.7 %, and spectrum registering step (RS) = 1 eV.

### A Fortuity of Large-Intensity Spontaneous Moving Line

Powders of Bi_2_O_3_, PbO, SrCO_3_, CaCO_3_, and CuO of special purity grade were used as starting material for solid-phase synthesis of the (Bi_1 − *x*_Pb_*x*_)_2_Sr_2_Ca_2_Cu_3_O_y_ ceramics. The value *х* has compounded 0.16–0.17. Powders were decarbonized at 840 °C for 10 h twice, then crushed, stirred, extruded, and subjected to solid-phase synthesis in the quasi-closed reactor at 840 °C within 300 h. Density and resistivity of the ceramic samples were 4.6 × 10^3^ kg/m^3^ and 5.5 × 10^−6^ ohm m at 300 К, respectively.

The samples of oxide ceramics were cracked, and the resulting fresh surfaces were investigated in the vacuum chamber of the spectrometer. In general, a lamellar structure and flocculent morphology with developed surfaces of grains and pores are characteristic for such objects [5]. It was found that the same surface texture is steady against the effect of electron irradiation applied in this investigation.

**Fig. 2:**
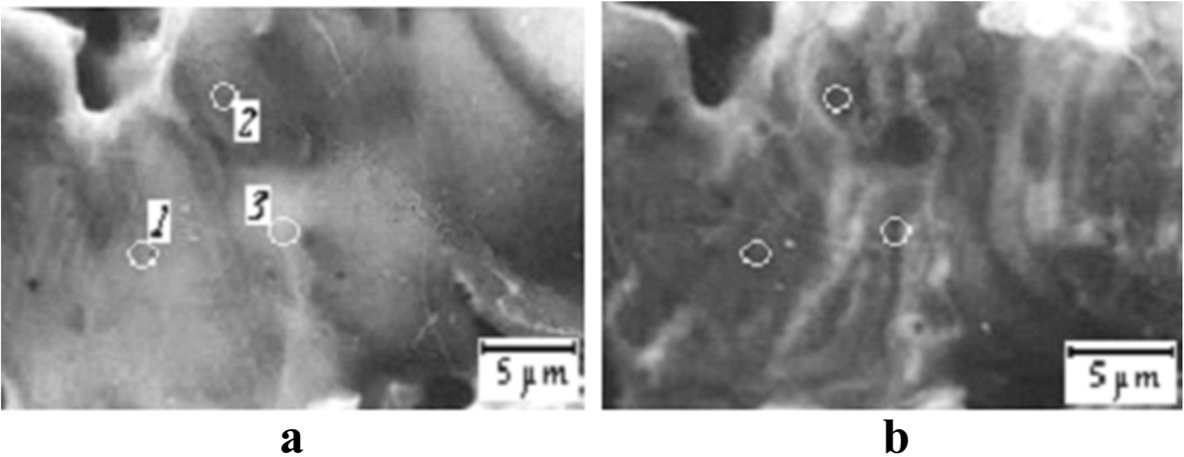
Processes of new surface producing in folds and grain boundaries at electron beam mild ionization. A presentable segment of large asymmetrical coagulum covered by the amorphous-like shell for the (Bi_1 − *x*_Pb_*x*_)_2_Sr_2_Ca_2_Cu_3_O_*y*_ ceramics (SEI): **a** fresh surface; **b** this surface after before-threshold electron beam exposition within 22 h (*E*_e_ = 5 keV; *i* = 0.08 А/mm^2^, RT); (*1*) to (*3*) were three microzones for local Auger analysis

But in some cases a raw of adjacent grains were integrated in rather large asymmetrical coagula covered by amorphous-like shells (Fig. 2a). The electron ionization promoted sharp destruction of the shells, disintegration of coagula, and formation of smaller steady grains. It is necessary to mark that the coagulum disintegration process has a threshold nature with the threshold on electron beam current density about 1 А/mm^2^ [3]. Above this threshold, the rough rapid changes in coagulum morphology took place.

In this work, the electron-raster researches were realized at a low-current primary electron beam: *E*_e_ = 10 keV; *i* ≤ 25 mA/mm^2^. That is why the influence of ionization at electron-raster researches was neglected (Fig. 2a). The electron spectra were registered also at the before-threshold values of beam current density (less than 0.1 А/mm^2^) which have not resulted in a sudden degradation of the ceramic grain coagula.

Three microzones for the analysis were selected in the range of one coagulum (Fig. 2a). They corresponded to two different under-surface grains (microzones 1 and 2) and to the boundary between adjacent grains (microzone 3).

The surface segregations of bismuth and strontium were watched for an amorphous-like shell in the regions of the under-surface grains (microzones 1 and 2). Probably, the smoothed coagulum morphology is connected with the ability of bismuth to form atomically tabulated films covering three-dimensional nanocrystals and microcrystals as it was found in [4].

But it was revealed that the shell of a coagulum in the region of microzone 3 was characterized by the relatively bright phase contrast (Fig. 2a), smaller intensity of all spectral lines, smaller concentration of oxygen, and larger concentrations of copper and residual carbon from SrCO_3_ and CaCO_3_. The slow replacement of the indicated elements on calcium and the formation of calcium oxide phase and corrugated shell morphology (Fig. 2b) took place in all the surface regions of the coagulum during the multi-hour electron ionization at the lowest (as possible for an Auger spectrum registration) beam current.

Morphology of the coagulum shell near microzone 3 transformed to the greatest degree, as the surface fold of dark phase contrast formed here (Fig. 2b). And the phenomenon of the spontaneous ML took place only in microzone 3 and, in the same time, did not realize for microzones 1 and 2. Particularly, this effect was already presented in [7].

Note that the electron beam ionization of the sample surface takes place in situ during the investigations. The conditions of registrations of electron spectra were selected experimentally for the purpose of avoiding a rough destruction of a surface and, simultaneously, obtaining a reasonable signal-to-noise ratio.

So, the energy of long-term electron beam exposition was *E*_e_ = 5 keV at the constant *i* = 0.08 A/mm^2^. The electron spectra were registered at the same parameters of the primary electron beam at *M* = 4 eV, *ΔЕ*/*Е* = 1.2 %, and RS = 1 eV. The vacuum level around the sample was near 2 × 10^−8^ Pa.

### Electron Beam Intense Ionization of Thin-Layer Systems with an Adsorbed Layer

**Fig. 3:**
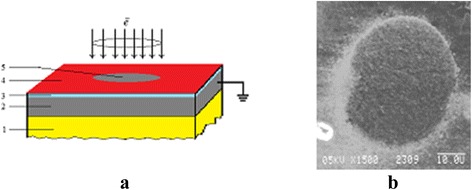
Realization of modeling non-equilibrium conditions for a thin-layer composite. **a** The scheme of a thin-layer composite system “inceptive adsorption layer *4* on the native oxide layer *3* (2 nm) on the metal film *2* (200 nm) on dielectric substrate *1*” and its defocused electron beam pulse ionization with active surface zone *5* after intense impact. **b** SEI image of active surface zone 5 with diameter nearly 50 μm after intense electron beam impact (*τ* = 8 ms, iron film)

Some samples of thin-layer systems “organic adsorption layer on the layer of native oxide (2 nm) on iron film (200 nm) deposited on dielectric substrate (mica)” (Fig. 3a) were investigated immediately after intense pulse electron beam ionization in one vacuum chamber [2] with the aim of inducing ML and its properties investigation.

Adsorption layers of surfactants were deposited on the surface of iron oxide from their solutions in isopropanol by the drip method [18]. For preparations of these thin-layer samples without an inceptive adsorption layer, a light sputtering of the iron oxide surface by argon ions was used.

The parameters of pulse electron beam irradiation when the ionization losses in solid prevail [13] were the following: *E*_ir_ = 25 keV, *i*_ir_ = 6 A/mm^2^, *τ* = 4–10 ms, a beam diameter of 50 μm, and an electrical grounding of iron film. After a similar intense pulse electron beam treatment, the active surface zone with a diameter nearly 50 μm appeared (Fig. 3b).

As results of a similar intense ionization, the following processes took place: electron-induced reactions between the adsorption layer and solid surface, desorption of molecular fragments in a vacuum, accumulation of a negative charge in a dielectric substrate, intensive grinding of metal film structure with dynamical change of a phase contrast from initial light to residual gray (Fig. 3b) and other related relaxation phenomena depended on the properties of metal film and primary adsorbed molecules [2, 18]. One of them is the appearance of electron-induced complex of MLs.

The electron spectra (AES and EIS) were registered in the range of active surface zone (Fig. 3) at *M* = 4 eV, *i* = 0.7 A/mm^2^, and RS = 0.1 eV.

The energy of the primary electron beam for achievement of ionization spectra was *E*_e_ = 1130 eV. The binding energy (BE) of electrons was determined with respect to a Fermi level for the spectrometer analyzer: BE = *E*_e_ − *E* (eV).

The vacuum level around the sample during pulse electron beam treatment and investigations was near 2 × 10^−8^ Pa.

The main accents in these investigations were in observation of initial stages and conditions of ML’s appearance, dynamics of their movement, conditions on finish stages when the MLs vanished, and finding the common regularities between spontaneous and induced MLs. The conditions of ML appearances were presented particularly above.

### Low-Intensity Moving Line at Argon Ion Sputtering of Steel Surface Layers

**Fig. 4:**
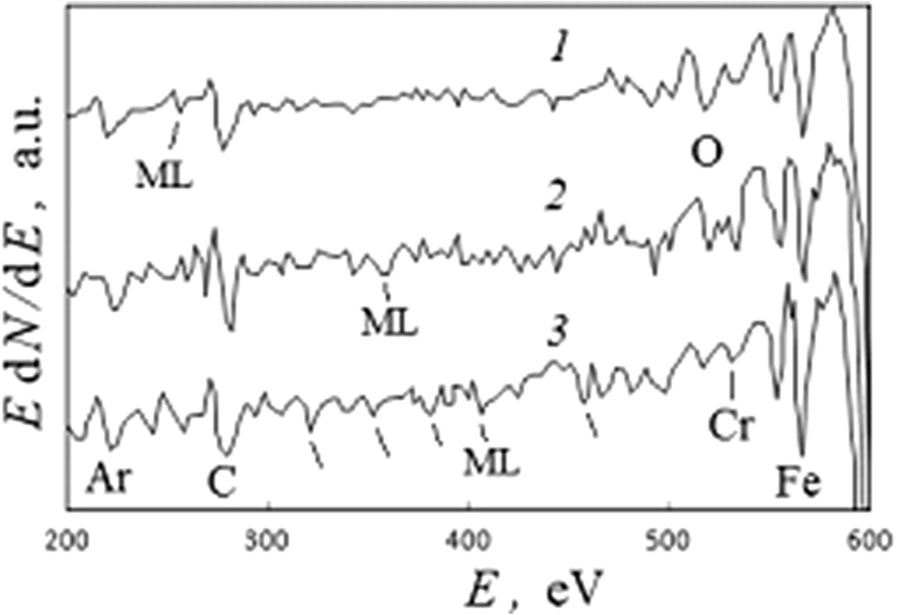
An incident of low-intensity moving line on spectra. The region of Auger spectra which presents the low-intensity ML for microzone 2 (Fig. 1) after argon ion sputtering within (*1*) 40, (*2*) 80, and (*3*) 100 min

**Fig. 5:**
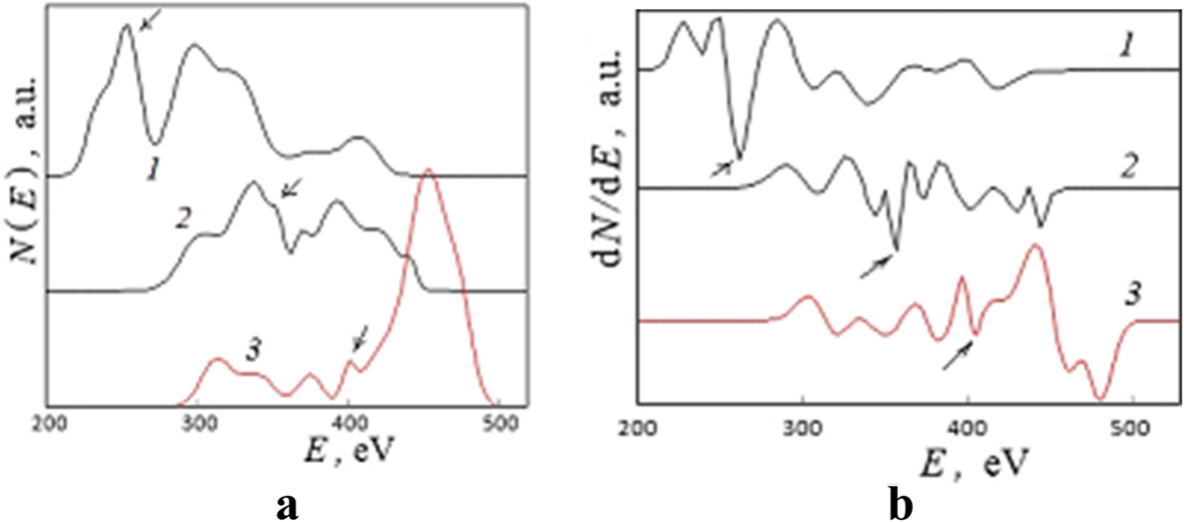
Changes in total scattering spectra connected with the low-intensity moving line. An incident of the low-intensity ML for microzone 2 (Fig. 1) after argon ion sputtering within (*1*) 40, (*2*) 80, and (*3*) 100 min: **a** direct and **b** differential spectra. The *pointers* indicate the ML positions in accordance to Fig. 4

During argon ion sputtering of zinc-enriched surface layers in microzone 2 (Fig. 1a), the ML which has a low intensity (the similar level to noise) was found on differential Auger spectra (Fig. 4). The integration of spectra (Fig. 4) allows to minimize a spectral noise and analyze the total scattering spectrum connected with low-intensity ML shape (Fig. 5a) after SBL procedure. The reverse numerical differentiation of the direct shapes *N*(*E*) with respect to energy *E* facilitates an analytical consideration (Fig. 5b).

**Fig. 6:**
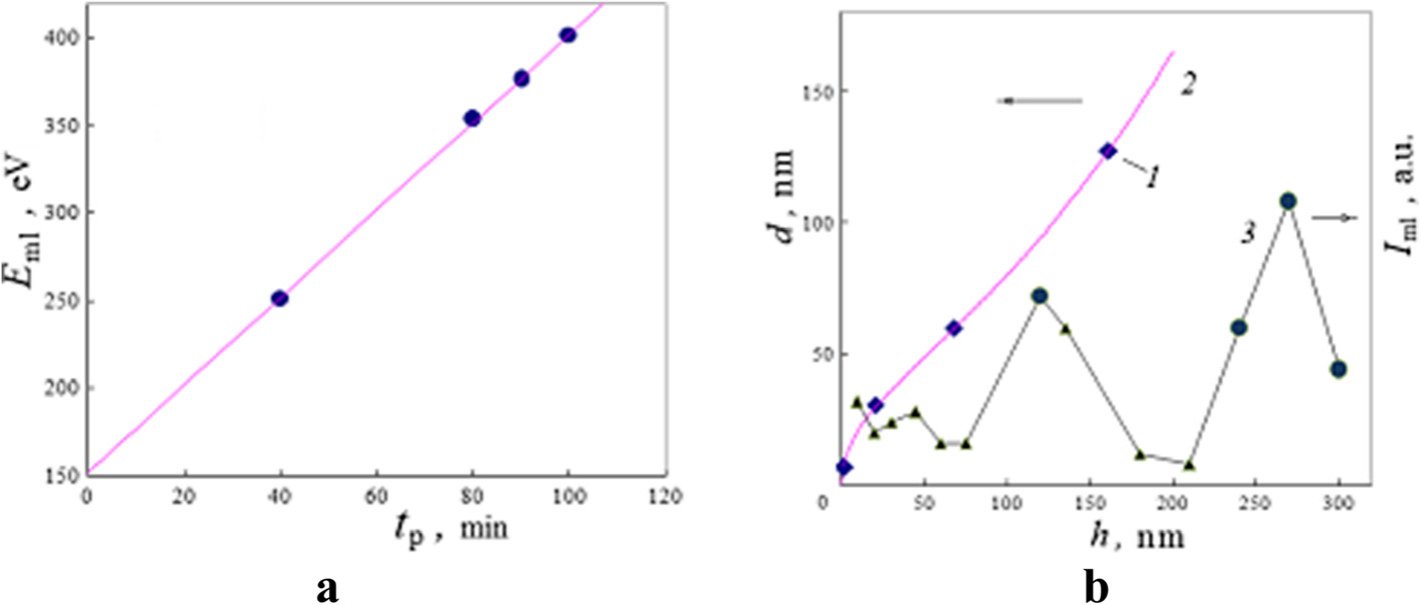
Dynamics and interlayer installation for processes which induce the low-intensity moving line. **a** The energy position *E*_ml_ of the ML for microzone 2 (Fig. 1) versus ion sputtering duration *t*_p_ and its linear extrapolation. **b** The correlations between (*1*) depth localization *h* of nanograins with diameter *d* [12], (*2*) extrapolative distribution *d*(*h*), and (*3*) the alteration of intensity *I*_ml_ of the ML in the surface layers

Because the marked ML energy positions, as it was found (Fig. 6a), are linearly proportional to the duration of ion sputtering other values of them may be calculated for all time moments (all points of the compositional depth profile) and values of ML amplitudes measure from all spectra registered in this experiment.

This line changed its position on the spectra in the direction of higher energies linearly in terms of ion sputtering with an effective rate of nearly RM ≃ 2.5 eV/min.

The correlation between dimensional distribution *d*(*h*) of nanograins within the surface layers, which was experimentally determined in [12], and ML intensity is illustrated in Fig. 6b. The points (1) in this figure indicate positions of central parts of the nanograins. The ML intensity essentially increases in regions between them.

So, the spectrum analysis (Fig. 5) shows that the ML phenomenon is connected with complex processes of electron scattering on locally charged surface layers. If taken into account a low intensity of this ML and noise, it may be found that any features of ML and related complex scattering spectrum are repeated in time for different energy positions of this line.

The correlative tendency in Fig. 6b is an example both of a local installation on the grain boundaries for processes which induce the ML phenomenon, and of their interconnection with general charge distribution in the surface layers.

### Large-Intensity Spontaneous Moving Line for Oxide Ceramic

**Fig. 7:**
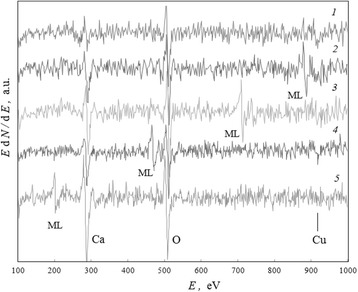
A fortuity of large-intensity spontaneous moving line on spectra. The region of Auger spectra for (Bi,Pb)_2_Sr_2_Ca_2_Cu_3_O_*x*_ ceramics which presents the ML for microzone 3 (Fig. 2) after low-intensity electron beam exposition (*E*_e_ = 5 keV, *i* = 0.08 A/mm^2^) for (*1*) 86, (*2*) 116, (*3*) 172, (*4*) 482, and (5) 1250 min

The intensive symmetrical line, connected with the ML phenomenon, had appeared in microzone 3 of the amorphous-like coagulum shell (Fig. 2a) for (Bi,Pb)_2_Sr_2_Ca_2_Cu_3_O_*x*_ ceramics after electron beam exposition *t* = 1.9 h in the regime of investigation (“A Fortuity of Large-Intensity Spontaneous Moving Line” section). This line could not be identified as a known spectral line for any chemical element (Fig. 7, spectrum 1) but imagined as very intensive satellite for main copper Auger line CuLMM (spectrum 2).

During subsequent 2 h, this line and its little satellites (Fig. 7, spectrum 3) were moving along the spectrum to smaller energies with the rate about RM ≃ 2.5 eV/min in respect to electron beam exposition time and had practically invariable intensity. The positions of spectral lines of calcium and oxygen remained stable in time, though their intensities increased. Note that after initial displacement of the ML, there was a decrease in intensities of the CuLMM Auger lines (spectra 2 and 3).

**Fig. 8:**
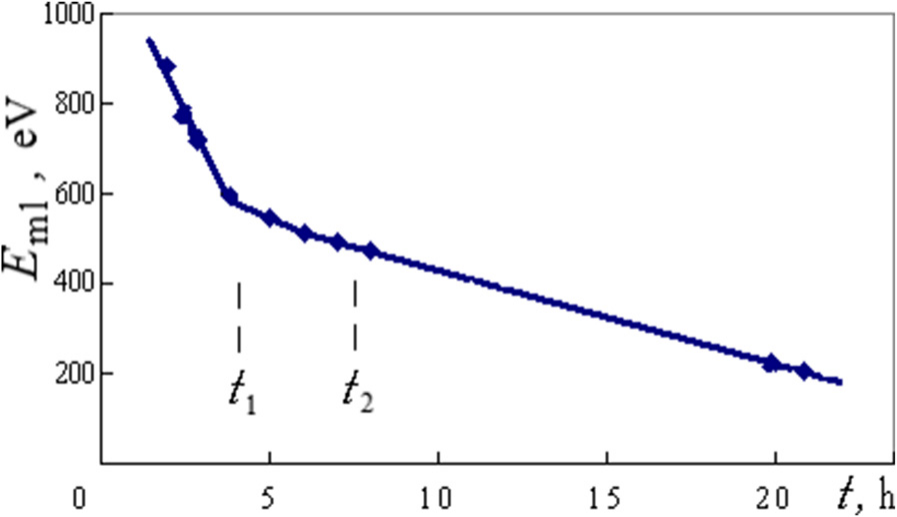
Dynamics in processes which induce the large-intensity moving line. The energy position *E*_ml_ of the ML for microzone 3 (Fig. 2) versus duration *t* of low-intensity electron beam exposition and its linear extrapolation: *t*_1_ and *t*_2_ indicate the time limits under a visible stage of surface fold formation

After achievement of maximum concentrations of calcium and oxygen, there were a continued restructuring of the coagulum and a creation of surface fold in the region where microzone 3 is registered (Fig. 2b). In this long-time period (Fig. 8, between *t*_1_ and *t*_2_ moments), the ML changed its position in the region of 550–450 eV and had not vanished; however, its moving rate along the spectrum decreased down to RM = 0.4 eV/min. Its intensity also decreased, but the intensities of the CuLMM lines, as an opposite, increased (Fig. 7, spectrum 4).

Within 19 h after the initial appearance at *E*_ml_ = *E*_0_ ≃ 887 eV (Fig. 7, spectrum 2), this ML had achieved a position near 200 eV (spectrum 5) and vanished when a new maximum of calcium concentration took place.

As it was found during ML displacement, the inflection on its linear time dependence (Fig. 8) is connected with the launch of global structural changes of the coagulum shell and the visible appearance of new surface fold (Fig. 2, microzone 3).

### Complex of Moving Lines Induced by Intensive Electron Beam Ionization

The phenomenon of electron-induced complex of MLs took place for all points of active surface zone after pulse ionization (Fig. 3b) but, as it was found [2, 18], depended essentially not only on experimental conditions and characteristics of thin-layer systems (in particular, thickness of iron films) but also on molecule properties and structure of the adsorption layer (Fig. 3).

#### The Moving Lines as a Reflex on Influencing of Inceptive Adsorption Layer

**Fig. 9:**
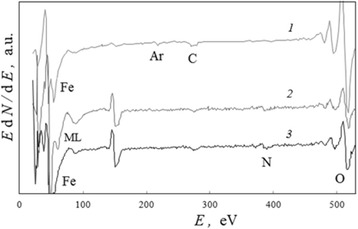
Changes in spectra of surface iron oxide induced by intense electron beam impact. The presentable region of Auger spectra for active surface zone (Fig. 3) in the case of thin-layer system without surfactant adsorption layer: (*1*) before, (*2*) immediately, and (*3*) through 5.4 min after pulse (*τ* = 8 ms) intense electron beam ionization

For the sample of a thin-layer system without the surfactant adsorption layer (Fig. 3a), i.e., for the system with a “clean surface” of iron oxide (Fig. 9, spectrum 1), the ML was induced at the region less than 100 eV through intense electron beam impact (spectrum 2) but lasted for a short time (spectrum 3).

**Fig. 10:**
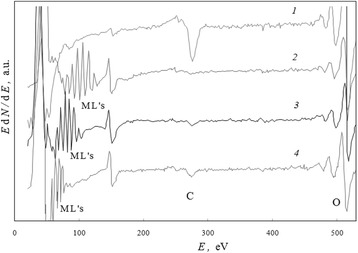
Changes in spectra of iron oxide with adsorbed isopropanol induced by intense electron beam impact. The region of Auger spectra which presents the electron-induced complex of MLs for active surface zone (Fig. 3) in the case of a thin-layer system with isopropanol inceptive adsorption layer: (*1*) before, (*2*) immediately, (*3*) through *t*_ml_ = 5.4 min, and (4) *t*_ml_ = 10.2 min after pulse (*τ* = 8 ms) intense electron beam ionization

The inceptive adsorption layer of alcohol (isopropanol) molecules (Fig. 10, spectrum 1) stimulates the appearance of complex of intensive MLs in an initial position up to *E*_ml_ = 125 eV (spectrum 2) within intense electron ionization. The prime spectra 2 → 3 → 4 (Fig. 10) were registered in discrete for about 4.9 min. The effective primary rate of the MLs was RM ≃ 4.6 eV/min. Let us note that the intensity of carbon line CKLL increases with displacement of MLs in the direction of lower energies.

**Fig. 11:**
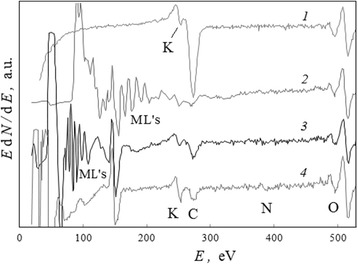
Changes in spectra of iron oxide with adsorbed stearate induced by intense electron beam impact. The region of Auger spectra which presents the electron-induced complex of MLs for active surface zone (Fig. 3) in the case of thin-layer system with KSt inceptive adsorption layer: (*1*) before, (*2*) immediately, (*3*) through *t*_ml_ = 5.4 min, and (*4*) *t*_ml_ = 10.2 min after pulse (*τ* = 8 ms) intense electron beam ionization

In the case of a surfactant such as potassium stearate (KSt), the inceptive adsorption layer (Fig. 11, spectrum 1) initiates a similar complex of MLs within the same intense electron beam impact but in a spectral region up to more than 200 eV (spectrum 2). Note also that with displacement of MLs to lower energies, the intensity of potassium line KLMM increases (Fig. 11).

As the spectra in Figs. 10 and 11 were registered with the same discrete time interval, we may conclude that an effective primary rate of MLs is nearly four times higher for the KSt inceptive adsorption layer (RM ≃ 18.1 eV/min) in comparison with the case of alcohol one.

So, the initial positions of the electron-induced MLs on the electron spectra after the same pulse intense ionization connect with the nature of inceptive adsorption layer, despite the fact that simultaneous electron-induced intense dissociation of molecular bonds, evaporation in vacuum, destructions in the adsorption and surface oxide layers, and fine grinding of iron film (Fig. 3b) took place. These complexes of MLs are composed of the original packets, a form which saved in main lines after their displacements on a spectrum. But the distance between separate adjacent lines in packets decreased with energy positions (Figs. 10 and 11).

#### The Complex of Moving Lines as a Reflex on Multiple Splitting of Ionized Electron Stages for Solid

**Fig. 12:**
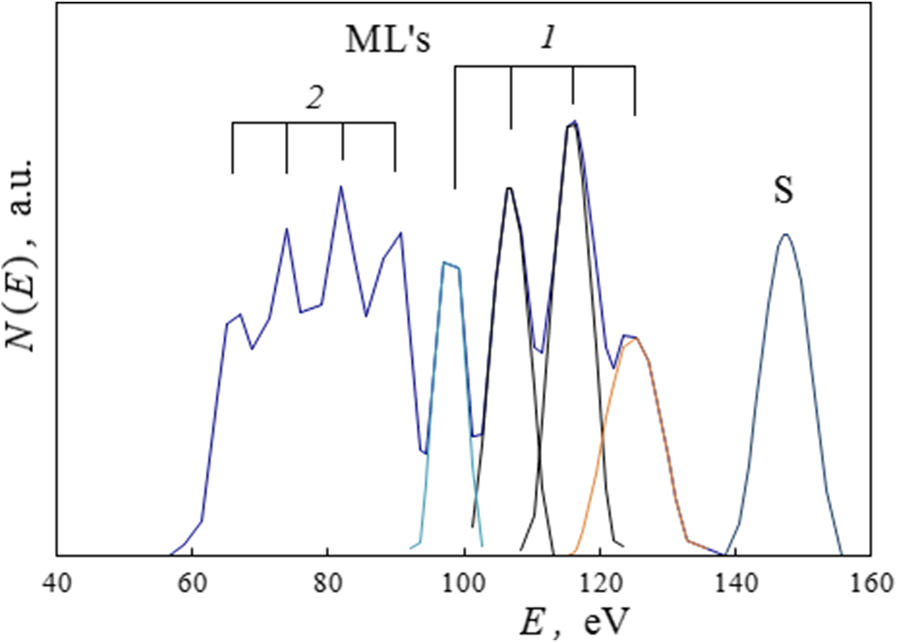
Detached spectrum for the initial complex of moving lines after intense electron beam impact. A spectrum *N*(*E*) of the MLs for the case of isopropanol inceptive adsorption layer, registered immediately after pulse intense electron ionization (Fig. 10, spectrum *2*): (*1*) indicates the higher energy packet 1 in the complex of MLs with four model component peaks; (*2*) indicates the lower energy packet 2 in the complex of MLs

A fine structure of the electron-induced complex of MLs was studied after integrations of registered spectra and SBL procedure. The direct spectrum *N*(*E*) of MLs at the initial moment of their appearance is presented as an example in Fig. 12.

It is found that by fitting, the complex of MLs consists of two packets of four different peaks which correspond to multiple MLs. The average distances between the adjacent peaks (*ΔE* = 9.0 eV in higher energy packet 1 and Δ*E* = 7.8 eV in lower energy packet 2 in Fig. 12) and widths of the peaks changed in the following times proportionally to energy position of MLs. The maximal observed distance achieved a value of *ΔE* = 15.8 eV (between the peaks at *E*_ml_ = 211.3 and 227.1 eV) in composition of packet 1 for the case of the KSt inceptive adsorption layer (Fig. 11, spectrum 2).

During ML displacements in the direction of lower energies (spectra 2 → 3 → 4 in Figs. 10 and 11), the effective distance between packets 1 and 2 as a whole (Fig. 12) decreased also and their peaks began to overlap. In addition, the lower energy part of packet 2 began to transform into a continuous spectrum.

**Fig. 13:**
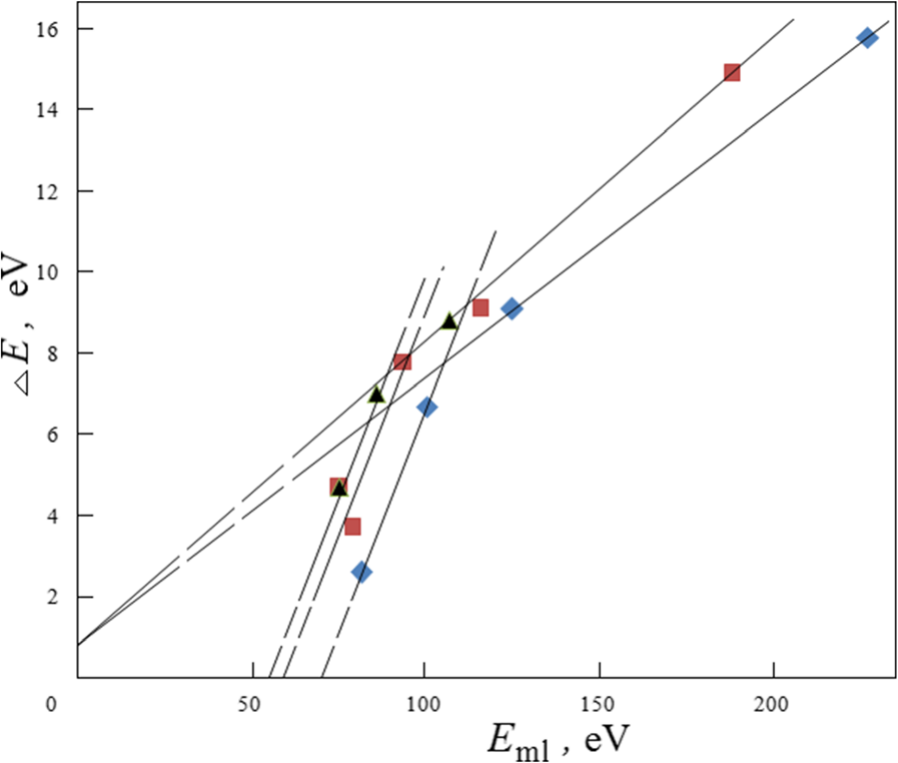
Changes in characters for complexes of moving lines as outcome for changeable local charge fields. The summary correlations between peak-to-peak distances *ΔE* and peak energy position *E*_ml_ on spectra for some peaks in the ML complex packet 1 (Fig. 12) in the cases of isopropanol and KSt inceptive adsorption layers

The summary correlations between peak-to-peak distances and peak positions on the spectra for some peaks in packet 1 are illustrated in Fig. 13. With displacements of different peaks to a zero energy of spectrum, the peak-to-peak distances linearly decrease with the next extrapolation to a value *ΔE* = 0.8 eV. This tendency illustrates the simultaneous effects of changeable electric fields from electron-induced charges on surface processes in the first time period after intense pulse ionization.

But during the next time period, the low-energy edge limited these peak displacements. In the region 90–100 eV, a moderation of peaks’ movement took place, and then these peaks slowly coincided with energy positions 55–70 eV and vanished.

The similar “ML multiple splitting” (Fig. 13, *ΔE* = 2.6–15.8 eV) is evidently a result of charge-induced splitting of solid core electron stages. In particular, these values are characteristic of “stable” satellite structures (*ΔE* = 3.9–14.7 eV) on *L*_2,3_ photoelectron spectra of 3d-transitional metals in various ionic compounds ([19], Table 52). As may be predicted for ions, the changeable resulting splitting for their electron stages takes place due to quadratic Stark effect in the field which varied with residual surface charge. Four peaks in one ML packet (Fig. 12) may indicate about Stark splitting for *M*_2_ or *M*_3_ ion stages (*n* = 3). So, the finishing peak-to-peak distances of *ΔE* = 3 ± 0.5 eV for the complexes of MLs (Fig. 13) may be caused by in situ final splitting ionized electron stages for iron film at the moment of ML disappearance.

It is known that the configuration interactions provide also any splitting of core electron stages for transitional elements [20]. Studying the influence of residual charge within a dielectric substrate, the ionization lines of iron Fe*L*_23_ were registered both in the center of the active surface zone (Fig. 3b) and far from it in the region of the initial sample surface for 1.7 h after the intense electron beam pulse. The ionization line shapes were studied after integration of the differential spectra, SBL, and normalization procedure with respect to same square for the line shapes.

**Fig. 14:**
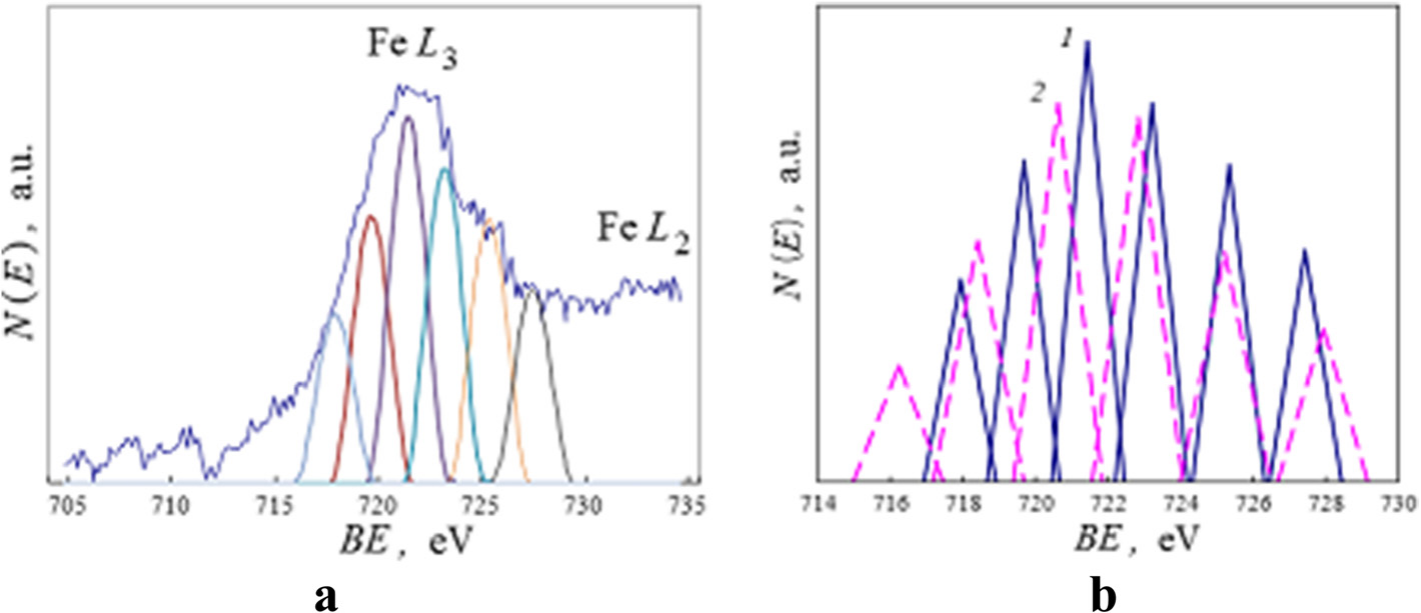
Long time differences for ionization Fe*L*_3_ line as outcome for local charge fields. Ionization spectra *N*(*E*) in the case of a thin-layer system with the isopropanol inceptive adsorption layer after 1.7-h interval from the intense electron beam impact: **a** Fe*L*_3_ line and its six fitting component peaks for initial sample surface, far from the active surface zone (Fig. 3); **b** the Fe*L*_3_ component peaks for (*1*) the initial sample surface and (*2*) the center of active surface zone; a base of schematic triangle is equal to a width by half-intensity of a peak

For the initial sample surface, it was found that the Fe*L*_3_ ionization line had the complex fine structure and its shape was fitted by six model component peaks with peak-to-peak distance 2.0 eV (Fig. 14a, b, continuous line 1). Simultaneously, in the central part of active zone, the same Fe*L*_3_ line and its six components still remained broader (with a width by half-intensity of the Fe*L*_3_ line from 5.0 to 6.1 eV) and the similar peak-to-peak distance was 2.5 eV (Fig. 14b, dotted line 2). Obtained results were confirmed also in the case of the KSt inceptive adsorption layer.

These analyses and results in general allow to associate the complexes of MLs both with initiated influence of active atoms of the adsorption layer, and with multiple splitting of iron partially ionized core electron stages which is a summary result of configuration interactions and Stark effect after pulse intense electron ionization of thin-layer metal-on-dielectric systems. So, the energy positions of MLs on the spectra connect with electron interatomic transitions between the energy levels of solid and adsorbed atoms and with initial intense increasing and next relaxation of local charges in surface layers and under-surface dielectric substrate.

### Dynamics of Moving Lines

The analysis of ML dynamics proves that these lines displace along the spectra in two time stages. The first of them is faster, depending much on the nature of the inceptive adsorption layer (Figs. 10 and 11). The second one (basic stage) is slow and associated with physical properties of solid surface layers and experimental conditions. Within the correctness and from discrete steps of the experimental observations, the ML displacements on the spectra were correlated with time and continuous with interval over 1 min.

**Fig. 15:**
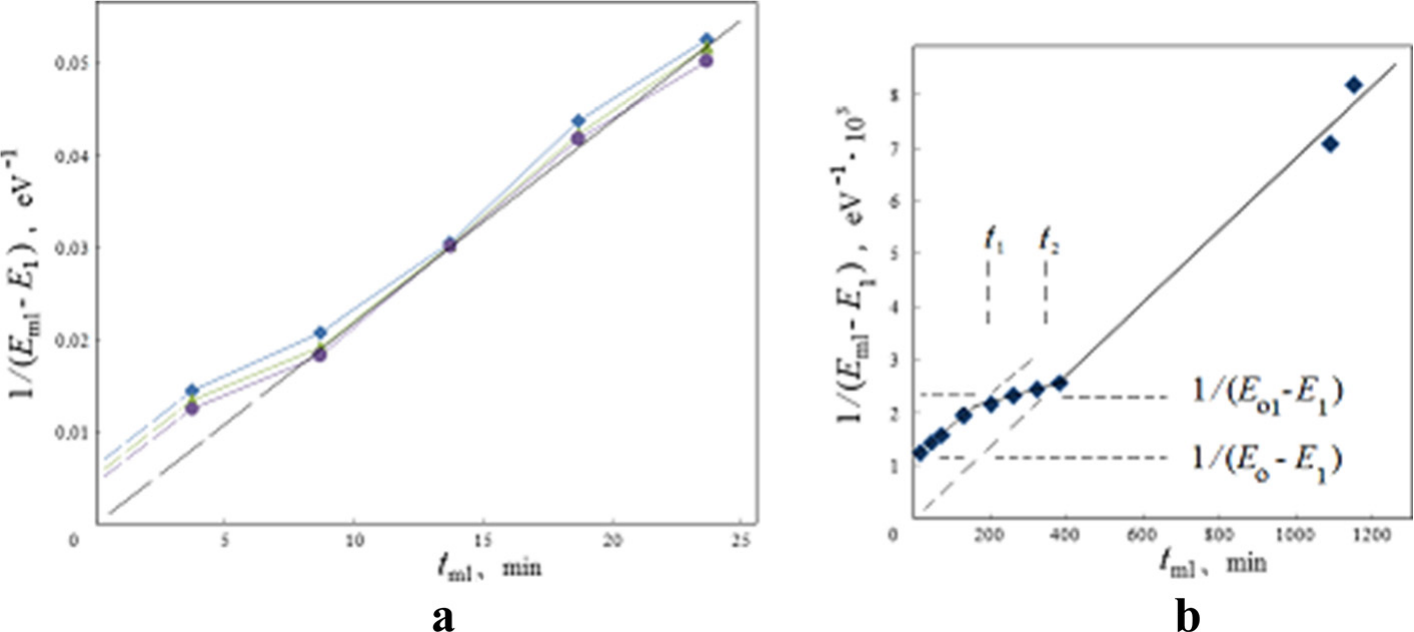
Dynamics of moving lines in coordinates for linear approximation. The time dependencies of energy positions of MLs in the coordinates of MD approach for: **a** three intensive peaks in packet 1 in the case of electron-induced complex of MLs (Fig. 12) and **b** spontaneous ML in the case of oxide ceramics (Figs. 7 and 8) where *t*_1_ and *t*_2_ indicate the time limits under a visible stage of surface fold formation

The time dependencies of ML energy positions are approximately good as linear in the coordinates of reverse energy versus ML time (MD approach). As an example, for electron-induced complex of MLs in the case of a thin-layer system with the alcohol inceptive adsorption layer (Fig. 10), three intensive peaks in packet 1 (Fig. 12) were shifted in accordance with a tendency represented in Fig. 15a. The same tendency which is found in Fig. 15b was characteristic also of the spontaneous ML in the case of oxide ceramic (Figs. 7 and 8). Let us note that the last material has also a good electrical conductivity as a whole (“A Fortuity of Large-Intensity Spontaneous Moving Line” section).

A comparison of various cases allows us to conclude about the proportionality of cotangent *D*_0_ of an angle of the basic line inclination (Fig. 15) with resistivity *ρ* of the sample and reverse value for electron beam density *i* and calculate from the dependencies in Fig. 15 as rough primary checking:

1 $$ {D}_0=\frac{\rho }{i}K, $$

where *K* ≃ 0.02 JVs/m^3^.

In both cases (Fig. 15), three characteristic values of energy play the key roles in dynamics of ML displacement on spectra: *E*_0_ for initial ML position, *E*_01_ for interstage transition, and *E*_1_ for final position, as the aim of ML displacement, and formally for achievement of linear MD approximation onto the first and second stages. In common, *E*_0_ and *E*_1_ are individual values for each peak (Fig. 12) in the packets of complex of MLs.

The type of time dependencies in Fig. 15 is subjected to a simple mathematical approximation:

2 $$ \begin{array}{l}{E}_{\mathrm{ml}}(t)={E}_1+\frac{D_1}{t_{\mathrm{ml}}+\frac{D_1}{E_0-{E}_1}}={E}_1+\frac{D_1}{t_{\mathrm{ml}}+\varDelta t},\kern1.5em 0\le {t}_{\mathrm{ml}}\le {t}_1,\\ {}{E}_{\mathrm{ml}}(t)={E}_{01},\kern2em {t}_1\le {t}_{\mathrm{ml}}\le {t}_2,\\ {}{E}_{\mathrm{ml}}(t)={E}_1+\frac{D_0}{t_{\mathrm{ml}}}={E}_1+\frac{\rho }{i{t}_{\mathrm{ml}}}K,\kern2em {t}_{\mathrm{ml}}\ge {t}_2,\end{array} $$

where *E*_ml_ is the current ML position on a spectrum; *t*_ml_ is the time from the initial moment of ML appearance; *t*_1_ and *t*_2_ correspond to start and finish, respectively, of the interstage transition (Fig. 15b); and *D*_1_ and *D*_0_ have a sense of “charge deed” in units of [energy × time] for initial and second basic time stages, respectively. Equation (1) is taken into account.

It is easily found that the values *t*_1_ and *t*_2_ may be presented in Equations (2) as dependencies on *E*_0_, *E*_01_, *E*_1_, *D*_1_, and *D*_0_, and therefore, the function *E*_ml_(*t*) is completely and clearly determined by these five parameters.

In common, from the experiments the interrelations *D*_1_ ≥ *D*_0_ and *D*_1_ = *kD*_0_ where *k* ≥ 1 were true. The equality *Δt* = *t*_2_ − *t*_1_ takes place when *D*_1_ = *D*_0_. This condition is close to the case of spontaneous ML (Fig. 15b), and period *Δt* = *t*_2_ − *t*_1_ is long enough and may be considered as separate transitional time stage.

### Discussion

Analysis of all the presented observations for the ML phenomenon on secondary electron spectra (long-term soliton form, satellite-like initial positions, continuous large displacement, etc.) allows us to prognosticate the MLs as reflexes on electron probe-induced strongly correlative intra- and interatomic and external charge processes in the region of a new solid interface which is created simultaneously (Fig. 2b, new surface fold; Figs. 3b and 6b, new grain boundaries). There are the electron probe ionization and screening of ionized core stages, an accumulation of space charge with the multiple splitting of core electron stages for intense enough probe impact, interatomic shake-up excitations in interfaces, and charge-induced changeable interatomic shifting of discrete electron levels. Similar correlations indicate that the ML process has a threshold character (for “spontaneous” ML also) and its activation energy must depend essentially on the surface properties of materials in original and ionized phases.

From another view, the processes of a new surface produced in folds and grain boundaries connect with appearance of the special state for surface atoms which supported the ML phenomenon. This special state turns up suddenly, and it is obviously a result of “prelaunch” electron probe-induced ionization and a critical increase of a surface charge on structural defects. In the next moment, after uprise, this state causes the simultaneous start of surface layers restructuration, recharging, and soliton-like ML reflections. The recharge process causes severely the long-term ML displacement on the spectra, but it does not crush their soliton form. This evidences the presence of a reverse potential barrier for ML disappearance. So, the special state for surface atoms should be complemented by the formation of quasi-stable virtual bound stages for such atoms in the ionized interface. A relative energy of each virtual stage must be changeable with the resulting field of local charges around the interface.

As known, charging effects on electron spectra which were different from a chemical shift, plasmons, ordinary satellite lines, etc., had been widely investigated earlier in non-conducting materials for a calibration procedure or an improvement of the registration of main spectral lines. For example, Johansson et al. [21] observed non-ordinary photoelectron spectral lines for mixed LiF and BN powders, explaining there as a result of non-uniform charging in the powder sample. Nemoshkalenko and Aleshin ([19], Fig. 180) illustrate photoelectron spectra for one surface region of catalyst in a charged state and after low-energy electron compensation. The additional “charge lines” on the spectra are similar to MLs at their initial spectral positions, and they disappeared after the charge compensation. But such “charge lines” are considered more in order of harmful experimental artifacts.

Nevertheless, the non-ordinary lines on the electron spectra are not “spurious” in common and may indicate about charge-induced hidden processes within interfaces for powder mixtures and catalysts. Abbamonte et al. [22] researched recently a dynamical reconstruction of the exciton in LiF crystal with inelastic X-ray scattering and shown that the exciton of Frenkel-like type is coherently delocalized over two unit cells (0.8 nm) with a lifetime of several femtoseconds. This excitation involves the interatomic transfer of an electron from Li^+^ to F^−^ ions, and it may explain the “anomalous” NK line in photoemission spectra for mixed LiF and BN powders [21]. As analyzed by Citrin [23], the interatomic electron transitions are beginning to be possible when intra-atomic ones are energetically or kinetically forbidden.

For conductive materials, the charge-induced interatomic shifting of electron levels is primarily connected with multiple ionized stages of atomic clusters absorbed in the interface. Many experimental results illustrate a significant sensitivity of interfaces to these stages. Averbukh et al. [24] argued that the interatomic electron correlation between cluster elements leads to a decay of intra-atomic vacancies by electron emission that causes often a disintegration of the ionized cluster.

In some spectral aspects (in particular, in the soliton-like form), the initial appearance of ML is similar to the revelation of known collapse effect for atomic electron orbit (or wave function collapse for exited electrons) [25, 26]. In particular, it may be also a result of pulse ionization (Fig. 11); Cowan [27] calculated that the collapse of 3d-electron is characteristic of potassium ion in configuration 3p^5^3d. Karaziya in his review [25] justifies that the electron collapse which may be realized as a charge-induced, ionization, or excitation (shake-up) effect in interfaces causes substantial reinforcement of the role of electrostatic interaction between ionized electron stages. It influences as on interatomic shifting in the interface as on probabilities of intra- and interatomic Auger transitions and cross sections of ionization processes. So the electron probe-induced electron collapse state in interfaces for any atoms can be seen as a realistic reason for “switching” of ML processes.

It is clear from the above that the physical causes and micromechanisms for the ML appearance-disappearance as spectral feature and the ML displacement on the spectra as a charge reflex on the surface layers (interface) restructuration must be mutually connected but are different. In changeable experimental conditions, the anomalous “charge lines” should be shifted essentially in time (excluding chemical shift) in relation to ordinary spectral lines, but presently, such specific researches by the methods of electron spectroscopy are unknown for us.

A dynamics of the ML displacement as a long-term relaxation process is similar for other non-equilibrium threshold phenomenon—photo-induced quasi-stable anisotropy (dichroism) in glass, especially for the As_2_Se_3_ and As_2_S_3_ materials, which was found by Zhdanov et al. [28]. From a numerical comparison for relative values, a character of dynamical decreasing for the difference in a dichroism value between saturated and current states of samples after a launch of a He-Ne laser intense pumping, as measured precisely by Hertogen et al. [29], is analogue to dynamical displacement of the MLs after intense electron beam ionization (Fig. 15a). Moreover, Kolobov et al. [30] illustrate the long-term residual broadening (similar to the one in Fig. 14b, splitting in the direction of smaller BE) for Se*M*_45_ core line (BE = 54.0–54.7 eV) on XPS spectra which is appeared only after laser pumping of the As_2_Se_3_ sample.

In general conception for the photo-induced dichroism, the quite intense linearly polarized laser beam induces the interatomic electron excitations (excitons) within structural defects of short-range order and orients these “lone atomic bonds” (as a result of supported excitation) for a long enough time accordingly to the direction of an electric field polarization. Electron probe-induced dichroism which was studied later by Shpotyuk et al. [31] in the As_2_S_3_ samples appears also in the region of optical absorption edge as a result of the creation of new defects in the form of “undercoordinated atomic pairs” oriented relatively to incident electron flow. The strong correlation between the processes of different phenomenological levels is pointed out as follows: induced interatomic electron excitation (electronic level) and changeable spatial orientation of atomic pairs (quasi-mechanical one).

It is also interesting to consider that for such complex effect, the cesium atoms have a transitional electron structure which makes possible the induced orbital collapse [25]. In connection to this, Molodtsov et al. [26] illustrate with the inverse photoemission spectroscopy that solid metallic cesium has no appearance on the effect that was explained by a conductive electron screening for electron probe-induced charge. Nonetheless, Ramesh and Ramaseshan [32] argued that in liquid cesium, the tunneling processes and dynamic conversion of 6*s* and 5*d* stages are causing the formation of a “virtual bound stage” which indicates the “continuous” electron collapsed phenomenon. The amount of collapsed atoms increases with pressure continuously. They have smaller atomic volume that is reflected on the sample resistivity.

The consideration of related phenomena allows us to predictably postulate the physical mechanisms for ML process as interatomic excitation of atoms in the interface with a rapid inducing of quasi-stable virtual collapsed stages and long-term orientation of the exited clusters with changeable interatomic distances.

Generally, we can find and identify five consecutive time periods for a common non-equilibrium state of the surface layers where ML phenomenon was revealed:

- The prelaunch stage during surface charge increasing to a large value (light phase contrast for actual surface regions, Figs. 1a and 2a)
- Stage 1 for a hidden restructuring process with appearance of “fast” MLs
- The transitional stage *t*_1_ − *t*_2_ for visible surface restructuring and contrast recharging (Fig. 15b)
- Stage 2 with final ML vanishing (dark or gray phase contrast as a final result of recharging for the surface regions)
- The last stage for a low charge surface after ML disappearance (Fig. 14b)

**Fig. 16:**
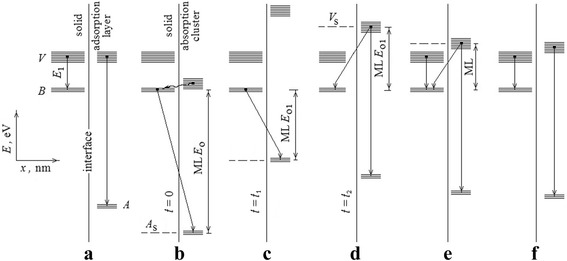
Provisional scheme for an interface installation of the moving line phenomenon. **a** A prelaunch stage. **b** The initial ML appearance, the start of hidden restructuring process. **c** A completion of stage 1 and the beginning *t*_1_ of visible surface restructuring. **d** The finishing *t*_2_ of the transitional stage and a starting of stage 2. **e** A final moment before vanishing of ML. **f** The last stage after ML disappearance. Denominations as ML*E*_0_ and similar which connect with ML energy positions are true if the energy of valence level *V* is zero. *Dotted lines* indicate the virtual levels *A*_s_ and *V*_s_ in solid

The inner interconnections between ionized atomic levels in the field of outer local charge, Auger electron spectral lines, and ML phenomenon may be now illustrated schematically as some “actual part” of the interface (Fig. 16). The scheme assumes every ML as Auger spectral line with changeable kinetic energy *E*_*A*s*BV*_ on the first stage and other changeable value *E*_*BV*s*V*_ on the second one which is connected with electron transitions through interface. This scheme explains the principled ability of ML appearance but must be evidently very different in various cases.

If *A* and *B* are the core electron levels for accented atoms in the future interface, their prelaunch states will be provisionally independent and characterized by AVV- and BVV-Auger lines (Fig. 16a). After electron probe-induced critical charging of surface layers, there are such overlapping processes as a sharp and strong dipping of the levels for ions which began to form absorbed clusters in a new interface, a probable resonance hybridization of BV stages and an induction of the changeable solid virtual level *A*_s_ in the interface, the initial interatomic A_s_BV-Auger relaxation, and simultaneous ML appearance with start energy *E*_0_ (Fig. 16b).

In whole, this is a new quality of an interface that causes the lingering recharging of surface layers at the electron flow influencing (stage 1) and the long-term relative lifting of ionized absorbed atom levels, the decreasing of *A*_s_-*B* difference, and ML displacement on the spectrum. Further decreasing of an outer local charge and (or) an ionization degree for absorbed atomic clusters is connected with the transition stage *t*_1_ − *t*_2_ (Fig. 15b) and activates the transitions from absorbed valence levels with appearance of both BV_s_V-(ML) and AVV-Auger lines, where *V*_s_ is other changeable solid virtual level (Fig. 16c, d). As consequence, there is an increase of known Auger lines for copper (Fig. 7, spectrum 4), carbon (Fig. 10), and potassium (Fig. 11) and simultaneously the changing (decreasing) of ML intensities. Next is slow reduction of surface charge (stage 2) which leads to the decreasing of *B*-*V*_s_-difference, ML displacement to final position (Fig. 16e), and its vanishing at the critically low value of cluster residual charge (Fig. 16f).

The phenomenological Equations (2) confirmed that the duration of ML phenomenon increases with *ρK* value which characterizes the scattering processes and, conversely, decreases with *it*_ml_ value for negative charge which is transferred in the incident electron flow through the surface layers. Formally, the first appearance and final vanishing of ML may be counted in the equations by turn-on for a charge deed value *D*_1_ in the moment *t*_ml_ = 0 and turn-off for a value *K* at finish.

If *D*_1_ = *kD*_0_ is true, the full period of ML manifestation can be introduced by an on-off switch of the constant *K*. The influence of the argon-positive ion beam on the ML position (Fig. 6a) may be counted by the on-off switch of the linear *i*_p_*t*_p_ addition in the value *K* where *i*_p_ and *t*_p_ are the ion current and summary duration of continuous ion sputtering of surface layers, respectively. Moreover, the “stable” ML position *E*_01_ during the transition stage *t*_1_ − *t*_2_ (Fig. 15b) may be presented some compensation for *i*_ml_*t* values for negative charge by temporal changes of the value *K*.

Presented results and the presumable mechanism of ML phenomenon are not ending but they are, rather, intended to focus on the effect and illustrate it in general. The similar moving lines (Fig. 16) may also be expected, for example, in the case of electron probe X-ray microanalysis but taking into account the specific conditions for their appearance. The prelaunch and initial conditions for the appearance of ML on electron spectra are of interest both for practice applications (for example, for the analysis of electronic and crystalline constitutions of surface layers) and for fundamental science. It is evidently that some nanomaterials will make possible now to concretize the ML physical nature more in detail. Any variants of interstitial atoms and surface intercalation processes seem likely to realize ML phenomenon. Availability of neighboring surfaces (extended interfaces, nanograin boundaries, multilayer films, charge condensed units, nano-capacitors, etc.) is essential also as implement.

## Conclusions

The analysis of experimental results presented in this work shows that the phenomenon of moving lines on electron spectra linked spatially and in time with a place of localization and a durability of the structural transformation of surface layers which produces a new surface square in folds, in grain boundaries, etc. These lines have sometimes the same intensities with “stationary” spectral lines of main elements.

It is assumed that the highly specific conditions of initial charging and the following recharging of surface layers under electron beam acting, which are taken for the profound structural transformation (without ruin) of solid surface, induce the variable interatomic shifting of electron stages in interface and cause, relatively to energy differences between these stages, the changeable energy positions of moving lines on electron spectra. Simultaneously, this effect is connected with more complex processes of electron scattering and charge distribution in the surface layers. As a result, the moving lines inform about a deeply hidden restructuring process and accompany visible surface transformations too.

It is found that the effect of moving lines is initiated in modeling conditions within simultaneous intense ionizations of thin metal films and high charging of a dielectric substrate. The nature of the inceptive adsorption layer in addition to the metal film determines the initial position of moving lines on the spectra.

## Abbreviations

AES: Auger electron spectroscopy
BE: binding energy of electrons determined relatively to Fermi level for spectrometer analyzer BE = *E*_e_ − *E* (eV)
*D*_1_ and *D*_0_: provisionally “charge deed” and cotangent of angle of linear MD inclination for stages 1 and 2, respectively, (J s)
*E*: kinetic energy of secondary electrons (eV)
*E*d*N*(*E*)/d*E*: differential mode of spectrum registration with cylindrical mirror analyzer modulation for analogue differentiation of direct electron spectra with respect to the energy E, and differential spectrum for secondary electrons, and suitably intensity on the differential spectra (a.u.)
*E*_e_: energy of incident electron beam (keV)
*E*_ir_: electron beam energy for intense ionization procedure (keV)
EIS: electron ionization spectroscopy
*E*_ml_: the energy position of ML on spectra which relates to kinetic energy of secondary electrons (eV)
GBC: general background distribution curve for inelastic electron scattering
*i*: electron beam current density for investigation (A mm^−2^)
*i*_ir_: electron beam current density for intense ionization procedure (A mm^−2^)
*I*_ml_: intensity of ML (a.u.)
*K*: “charge deed” core value (JVs m^−3^ = A^3^s^2^ m^−3^)
KSt: potassium stearate
*M*: modulation amplitude (eV)
MD: time dependency of (*E*_ml_(*t*) − *E*_1_)^−1^ type for energy position of ML
ML: the soliton-like moving line on electron spectrum
MLs: moving lines
*n*: principle quantum number
RM: effective rate of ML displacement on spectra (eV/min)
RS: spectrum registering step (eV)
RT: room temperature (°C, K)
SBL: procedure for subtraction of GBC and Auger line shapes from the spectrum of secondary electrons
SEM: scanning electron microscopy
*t*: time of electron beam exposition during investigation (min, h)
*t*_ml_: time period after initial appearance of MLs (min, s)
*t*_p_: the term of ion sputtering duration (min)
*ΔE*: average distance between adjacent peaks in a packet of ML complex and average value for splitting within the satellite structure of electron spectra (eV)
*Δt*: effective time period for highly ionized general state of surface layers (min, s)
Δ*Е*/*Е*: the mode constant ratio (%)
*τ*: pulse duration for intense electron beam ionization (ms)

## References

[1] Bandaru PR, Yamada H, Narayanan R, Hoefer M (2015). Charge transfer and storage in nanostructures. Materials Science and Engineering: R.

[2] Mischuk ОA (1992). The role of organic films in thermal processes induced by electron beam at the oxidized metallic surface. Proc. 182 Meet. Electrochem. Society, Toronto, October 11-16, 1992.

[3] Vasylyev MA, Bakuntseva MV, Mishchuk OA (1994). Threshold change of morphology and elemental composition of surface of (Pb_x_Bi_1-x_)_2_Sr_2_Ca_2_Cu_3_O_y_ceramics at intensive irradiation with electrons. Metallofiz Nov Tekh+.

[4] Goriachko A, Shchyrba A, Melnik PV, Nakhodkin MG (2014). Bismuth growth on Ge(111): evolution of morphological changes from nanocrystals to films. Ukr J Phys.

[5] Gorbyk PP, Chuiko AA, Bakuntseva MV (2003). Systems with a developed surface and phase transitions “conductor—high-temperature super conductor, semi-conductor—metal, dielectric—super ion conductor”.

[6] Khadzhai GY, Vovk NR, Vovk RV, Savich SV, Kislitsa M, Kotvitskaya KA (2015). Effect of high pressure on conductivity in the basal plane of Y_1-x_Pr_x_Ba_2_Cu_3_O_7-δ_ single crystals lightly doped of praseodymium. Functional Materials.

[7] Mishchuk OA, Korostil IA (2002). A peculiarity of electron spectra of the HTSC-ceramics at electron irradiation inducing transformation of grains. Abst. EMAS 2002: 5^th^ reg. workshop on electron probe microanalysis of materials today—practical aspects, Szczyrk, Poland, May 22-25, 2002. Szczyrc.

[8] Gnecco E, Meyer E (2015). Fundamentals of friction and wear on the nanoscale. 2nd ed. (NanoScience and Technology Ser.).

[9] Braun OM, Naumovets AG (2006). Nanotribology: microscopic mechanism of friction. Surface Science Report.

[10] Calcagnotto M, Adachi Y, Ponge D, Raabe D (2011). Deformation and fracture mechanisms in fine- and ultrafine-grained ferrite/martensite dual-phase steels and the effect of aging. Acta Mater.

[11] Mittemeijer EJ, Somers MAJ (2015). Thermochemical surface engineering of steels (Woodhead Publ. Ser. in Metals and Surface Engineering, No. 62).

[12] Міshchuk ОA, Теlеmkо ОV (2015). Gradient relaxation of steel surface in zone of Hertzian contact at friction. Metallofiz Nov Tekh+.

[13] Goldstein JI, Yakowitz H (1975). Practical scanning electron microscopy.

[14] Briggs D, Seach MP (1983). Practical surface analysis by Auger and x-ray photoelectron spectroscopy.

[15] Koval IF, Lysenko VN, Melnyk PV, Nakhodkin NG, Nakhodkin NG (1989). Atlas of ionization spectra.

[16] Johnson KL (1985). Contact mechanics.

[17] Kostetsky BI (1992). The structural-energetic concept in the theory of friction and wear (synergism and self-organization). Wear.

[18] Mishchuk OA, Kobylinska IF (1996). The influencing of organic molecules on changing of a surface concentration of oxygen at high-speed heating of metal. Kataliz i Neftekhimiya (Catalysis and Petrochemistry).

[19] Nemoshkalenko VV, Aleshin VG (1979). Electron spectroscopy of crystals.

[20] Carlson TA (1975). Photoelectron and Auger spectroscopy.

[21] Johansson G, Hedman J, Berndtsson A, Klasson M, Nilsson R (1973). Calibration of electron spectra. J Elec Spec Rel Phenomena.

[22] Abbamonte P, Graber T, Reed JP, Smadici S, Yeh C-L, Shukla A (2008). Dynamical reconstruction of the exciton in LiF with inelastic x-ray scattering. Proc National Acad Sci USA (PNAS).

[23] Citrin PH (1974). Interatomic Auger transitions: a survey. J Elec Spec Rel Phenomena.

[24] Averbukh V, Demekhin PV, Kolorenč P, Scheit S, Stoychev SD, Kuleff AI (2011). Interatomic electronic decay processes in singly and multiply ionized clusters. J Elec Spec Rel Phenomena.

[25] Karaziya RI (1981). The collapse of orbit of exited electron and peculiarities of atomic spectra. Uspekhi Fizicheskih Nauk (Achievements of Physical Sciences).

[26] Molodtsov SL, Laubschat C, Kaindl G (1994). Collapse of the 4f wave function in cesium. Phys Rev B.

[27] Cowan RD (1968). Theoretical study of p^m^-p^m-1^l spectra. J Opt Soc Am.

[28] Zhdanov VG, Kolomiets BT, Lyubin VM, Malinovskii VK (1979). Photoinduced optical anisotropy in chalcogenide vitreous semiconducting films. Phys Stat Sol A.

[29] Hertogen P, Tikhomirov VK, Adriaenssens GJ (1999). Photoinduced dichroism in chalcogenides: influence of temperature and light intensity. J Non-Crys Solids.

[30] Kolobov AV, Kostikov YP, Lantratova SS, Lyubin VM (1991). Investigation of photo-structural transformation in chalcogenide vitreous semiconducting films by the method of photoelectron spectroscopy. Fizika tverdogo tela (Solid State Physics).

[31] Shpotyuk OI, Balitska VO, Vakiv MM (1998). Effect of electron-induced dichroism in vitreous As_2_S_3_. J Non-Crys Solids.

[32] Ramesh TG, Ramaseshan S (1972). Electron collapse and the resistivity of liquid cesium. Physics Letters A.

